# Consensus clustering with missing labels (ccml): a consensus clustering tool for multi-omics integrative prediction in cohorts with unequal sample coverage

**DOI:** 10.1093/bib/bbad501

**Published:** 2024-01-10

**Authors:** Chuan-Xing Li, Hongyan Chen, Nazanin Zounemat-Kermani, Ian M Adcock, C Magnus Sköld, Meng Zhou, Åsa M Wheelock, U-BIOPRED study group

**Affiliations:** Respiratory Medicine Unit, Department of Medicine Solna & Centre for Molecular Medicine, Karolinska Institutet; School of Biomedical Engineering, Wenzhou Medical University, Wenzhou, China; National Heart and Lung Institute, Faculty of Medicine, Imperial College London, London, United Kingdom; Data Science Institute, Imperial College London, London, United Kingdom; National Heart and Lung Institute, Faculty of Medicine, Imperial College London, London, United Kingdom; Data Science Institute, Imperial College London, London, United Kingdom; Respiratory Medicine Unit, Department of Medicine Solna & Centre for Molecular Medicine, Karolinska Institutet; Department of Respiratory Medicine and Allergy, Karolinska University Hospital Solna, Stockholm, Sweden; School of Biomedical Engineering, Wenzhou Medical University, Wenzhou, China; Respiratory Medicine Unit, Department of Medicine Solna & Centre for Molecular Medicine, Karolinska Institutet; Department of Respiratory Medicine and Allergy, Karolinska University Hospital Solna, Stockholm, Sweden

**Keywords:** multi-omics integration, consensus clustering, missing labels, unequal sample coverage, predictive labels

## Abstract

Multi-omics data integration is a complex and challenging task in biomedical research. Consensus clustering, also known as meta-clustering or cluster ensembles, has become an increasingly popular downstream tool for phenotyping and endotyping using multiple omics and clinical data. However, current consensus clustering methods typically rely on ensembling clustering outputs with similar sample coverages (mathematical replicates), which may not reflect real-world data with varying sample coverages (biological replicates). To address this issue, we propose a new consensus clustering with missing labels (ccml) strategy termed ccml, an R protocol for two-step consensus clustering that can handle unequal missing labels (i.e. multiple predictive labels with different sample coverages). Initially, the regular consensus weights are adjusted (normalized) by sample coverage, then a regular consensus clustering is performed to predict the optimal final cluster. We applied the ccml method to predict molecularly distinct groups based on 9-omics integration in the Karolinska COSMIC cohort, which investigates chronic obstructive pulmonary disease, and 24-omics handprint integrative subgrouping of adult asthma patients of the U-BIOPRED cohort. We propose ccml as a downstream toolkit for multi-omics integration analysis algorithms such as Similarity Network Fusion and robust clustering of clinical data to overcome the limitations posed by missing data, which is inevitable in human cohorts consisting of multiple data modalities. The ccml tool is available in the R language (https://CRAN.R-project.org/package=ccml, https://github.com/pulmonomics-lab/ccml, or https://github.com/ZhoulabCPH/ccml).

## INTRODUCTION

As clinical research advances, omics data has become an indispensable tool for understanding complex diseases such as cancer, asthma and chronic obstructive pulmonary disease (COPD). By providing a comprehensive understanding of the underlying molecular mechanisms and interactions [1–3], omics data have enabled a deeper exploration of the molecular landscape of these ailments. However, the integration of these heterogeneous and complex data types poses a significant challenge [3–5]. Integrative analysis of multiple omics data types offers a more exhaustive and precise understanding of the underlying biological processes, leading to the development of more effective and personalized treatment strategies.

Unsupervised integrative clustering of multiple omics datasets from the same cohort to improve the statistical power to detect previously unknown subgroups of patients represents an increasing trend in data analysis, especially in precision medicine efforts of complex and/or chronic diseases. Several integrative methods such as Similarity Network Fusion (SNF) [6] and iCluster [7] have been developed for this purpose. These methods exploit the inherent relationships between different molecular entities and use them to integrate omics data into a common network representation. However, these methods still face challenges in handling missing data and determining the optimal clustering result [2].

We have previously shown that the integration of multiple omics datasets can significantly enhance the statistical power to detect subgroups in small clinical cohorts [2]. However, with an increase in the number of omics datasets, missing samples become more likely. This can result in repeated predictions with unequal sample coverages (missing labels) when clustering based on combinations of different multi-omics datasets. Consensus clustering is a suitable approach for ensembling these different clustering labels [8–12]. However, in the conventional consensus clustering method, each repeated subsampling and clustering has equal sample coverage, which results in an ensemble procedure that treats each clustering equally, leading to bias.

To overcome the limitations posed by missing data in multi-omics integration analysis, we have developed consensus clustering with missing labels (ccml). This is an extended consensus clustering tool for multi-omics integrative prediction with unequal sample coverages. Ccml introduces a new adjusted consensus weight (CW) among repeated clustering with unequal sample coverage as an extension of the current consensus clustering method. The ccml tool is available in the R language at CRAN (https://CRAN.R-project.org/package=ccml) or at GitHub (https://github.com/pulmonomics-lab/ccml or https://github.com/ZhoulabCPH/ccml), and extends the new functionality and visualizations of the R-package ConsensusClusterPlus of traditional consensus clustering [8]. Our proposed ccml tool is a valuable downstream tool for algorithms such as SNF [6] and robust clustering of clinical data and can help researchers to overcome the limitations posed by missing data in multi-omics integration analysis. Evaluation of the ccml package using the Karolinska COSMIC cohort demonstrates that the method can be used to facilitates multi-omics integration using data platforms with up to 60% missing data with equally robust—or improved—similarity in co-clustering of subjects compared to traditional methods. As such, ccml may help overcome the limitations posed by missing data, which is inevitable in human cohorts consisting of multiple data modalities.

## SOFTWARE FEATURES

The input format for ccml is a user-defined data matrix and customizable options. The data matrix represents a collection of multi-omics integrative clustering runs (in columns) for a specific set of samples (in rows). For example, this could be the output from Similarity Network Fusion and Spectral Clustering (SC) of all *n*-omics combinations (where *n* ≥ 5). The user can specify the maximum number of clusters (maxK) for clustering. The output includes stability evidence for a given number of permutations (*nperm*), cluster assignments, and adjusted or normalized consensus weights (NCW). The output comprises R data objects, text files and graphical plots.

### Algorithm

Ccml improves upon the original consensus clustering algorithm by addressing the issue of missing labels caused by unequal sample coverages. The rational for introducing NCW is grounded in our previous research, where we demonstrated that the integration of multiple omics datasets significantly enhances the statistical power to detect subgroups in small clinical cohorts [2]. Specifically, we found that multi-omics data fusion improves the accuracy of unsupervised molecular classification in the presence of confounding factors, such as smoking. For instance, as shown in our previous work [2], the mean accuracy of group prediction increased linearly with the number of omics datasets (*n*-tuple), from a mean accuracy of 0.28 for single-omics platforms to 0.90 for the septuple omics networks when using the label propagation approach. Moreover, septuple omics integration decreased the required subgroup size from *n* = 30 for single omics to *n* = 6 for septuple omics at the 95% accuracy level [2]. However, the frequent issue with missing values in multi-omics investigation of clinical cohorts hamper the utility of these methods, as the original CW algorithm lacks robustness toward missing data. These observations underpin the fundamental hypothesis driving the development and utilization of NCW.

**Figure 1:**
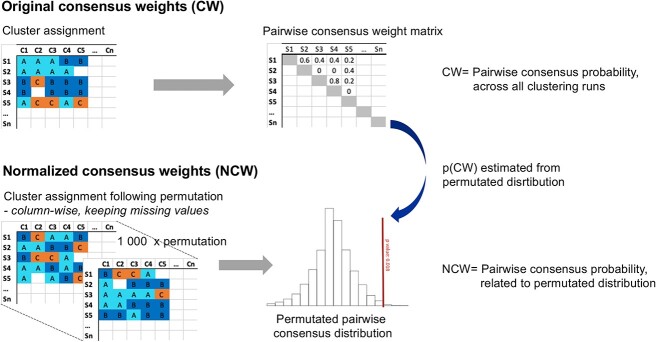
The input of the NCWs algorithm is a matrix is the same as for the original CWs algorithm, where each row is a subject in the cohort and each column is a clustering result from each of the possible data modality combinations. For the exemplification using the COSMIC cohort, the input would consist of the 607 possible networks generated from all possible combinations of the 9 available omics datasets. In the original CW, a sample matrix is generated where each cell reports the fraction of the total number of clustering iterations where the sample pair is clustered together, the pairwise CW matrix, with range 0–1 (upper panel). The input cluster assignment matrices are permutated column-wise, while keeping the missing data points (lower panel). The CW calculated as described above is then inserted into the permutated pairwise consensus distribution, and the probability (*P*) of the CW coming from the permutated distribution is calculated. NCW is calculated as 1 − *P* of the distribution.

To address these issues, ccml introduces the concept of NCW, a novel adjustment to the consensus clustering algorithm designed to handle varying sample coverages across different data modalities. The input of the NCW algorithm is a matrix as with the original CW algorithm, where each row is a subject in the cohort, and each column is a clustering result from each of the possible data modality combinations. For the exemplification using the COSMIC cohort, the input would consist of the 607 possible networks generated from all possible combinations of the 9 available omics datasets (available upon request). In the original CW, a sample matrix is generated where each cell reports the fraction of the total number of clustering iterations where the sample pair is clustered together, the pairwise CW matrix, with range 0–1 (Figure 1, upper panel). However, this approach is based on the assumption that all data points are available for all samples/subjects, which is generally not the case in clinical cohorts. If utilized as is, the classical CW results in a bias for sample pairs with a high degree of data missingness, as the missingness itself will be calculated as similarity.

The challenge arises due to missing data in specific modalities for different subjects, resulting in variations in the number of available *n*-tuple omics combinations and sample sizes (*n*) among subjects in each dataset. To address these variations, ccml calculates the NCW by evaluating the significance (*P*-value) of the one-sided empirical Cumulative Distribution Function using *nperm* permutations of the input matrix, with permutations performed within each column. In brief, the input cluster assignment matrices are permuted column-wise, while keeping the missing data points (Figure 1, lower panel). The CW calculated as described above is then inserted into the permutated pairwise consensus distribution, and the probability (*P*) of the CW coming from the permutated distribution is calculated. NCW is calculated as 1 − *P* of the distribution. This adjustment effectively normalizes the CWs (calculated using the R-package diceR [13]), accounting for the unequal sample coverages present in the data. The significance testing enables us to distinguish the confidence associated with clustering, even when using the same original weight (e.g. 0.3) across different omics combinations, such as 5-omics and 9-omics.

The final consensus cluster assignment is derived from a consensus clustering of the NCW matrix using ConsensusClusterPlus with a user-specified clustering algorithm [8]. To estimate the stability of permutations and ensure the robustness of the results, the tool calculates the squared Euclidean distance between NCW at regular intervals, typically every 1000 steps.

By introducing NCW into the consensus clustering process, ccml offers a valuable tool for multi-omics integration analysis, aligning with the findings of our previous work and allowing researchers to account for the challenges posed by varying sample coverages and missing labels in complex biomedical datasets.

### Output and visualizations

Ccml produces both numerical results and graphical plots that extend the capabilities of the ConsensusClusteringPlus package [8]. The output of the main function is a list vector consisting of three items: (1) ncw—a matrix of NCWs; (2) fcluster—a list of length maxK, where each element is a list containing consensusMatrix (numerical matrix), consensusTree (hclust) and consensusClass (consensus class assignments); and (3) icl—a list of two elements: clusterConsensus and itemConsensus corresponding to cluster-consensus and item-consensus.

**Figure 2:**
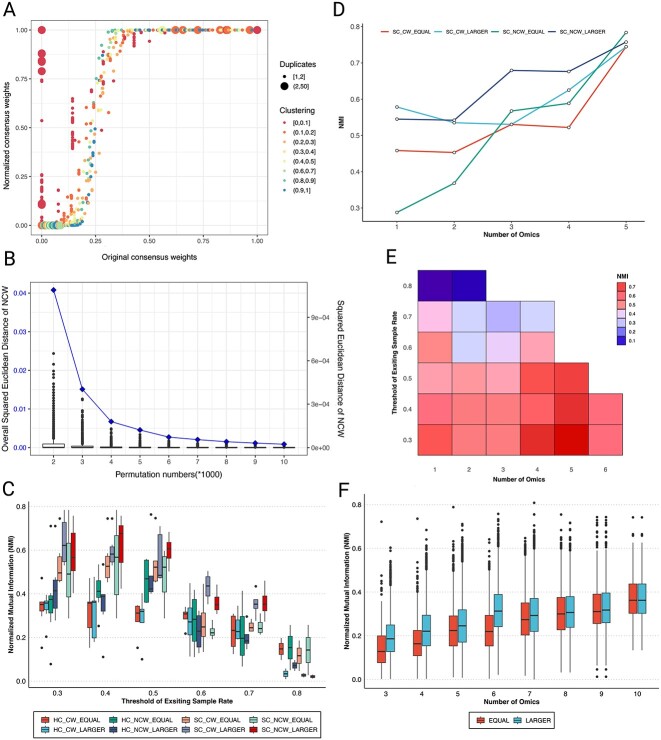
Example applications of multi-omics integrative clustering by means of ccml in two distinct cohorts: The Karolinska COSMIC cohort investigation COPD (**A**–**E**) and the U-BIOPRED cohort investigating severe asthma (**F**). (**A**) Correlation of the NCWs (*y*-axis) and original CWs (*x*-axis) strategies for omics combinations with existing sample rates of ≥40%, and networks consisting of five or more omics platforms. NCW (*y*-axis) provides a more relevant indication of the similarity in the clustering of subjects between iterations, as the weights are based on the significance level compared to a permutated background level of pairwise clustering (see Figure 1). Colors indicate *n*-omics platforms included, with red representing 10% or less of the platforms included, and green representing 90% or more of platforms included. (**B**) Stability of permutation numbers (*nperm*) for the NCW algorithm, using sample pairs with ≥40% existing sample rates for the respective omics combination, and equal to or larger than five omics data modalities available. (**C**) Boxplot displaying the accuracy of group prediction, estimated as normalized mutual information (NMI) between the true label of the COSMIC cohort and predictive clusters generated by (i) two different clustering methods, SC and hierarchical clustering (HC), (ii) two weights NCW or CWs, and (iii) the two omics integration strategies EQUAL or LARGER. Data shown as median with interquartile range. (**D**) The accuracy of group prediction, estimated as the normalized mutual information (NMI) compared to the defined cohort subgroups, as a function of *n*-omics platforms integrated. SC was performed in combination with NCW or CW, and EQUAL or LARGER strategies, respectively. Whereas all strategies merge at 5-tuple omics integration, NCW with the LARGER strategy provides more robust NMI at 3–4-tuple integration. (**E**) Heatmap illustrating the predictive accuracy (NMI; heatmap with dark red=1 and dark blue =0) in relation to the number of omics platforms included (*x*-axis) and the threshold for missingness (existing sample rate, *y*-axis) when using SC combined with NCW and the LARGER strategy. For the dataset at hand, a higher number of omics datasets with a more permissive threshold for missing data provides a better predictive power than that of fewer data platforms with a lower threshold for missing data. (**F**) Boxplot (median with interquartile range) of NMI accuracy between final ensembled predicted label output from ccml with predictive clusters by SC of each multi-omics combinations in EQUAL or LARGER strategies, respectively, using the U-BIOPRED cohort. The overall discrepancy in NMI seen between panels (**A**) and (**F**) are a reflection of the drastically different inclusion strategies and data missingness of the two cohorts.

PlotCompareCW illustrates the effect of original and NCWs in Figure 2A. The size of the colored portion that appears in the clustering runs indicates the number of duplicate samples. The point graph distribution of horizontal and vertical coordinates shows the weight distribution of different methods. The stability plot shows the result of calculating and estimating the stability of the permutation number for NCW (Figure 2B). Each box graph represents the Euclidean distance of non-null values in the original data after *nperm* * 1000 permutations. The line chart represents the sum of Euclidean distances after every 1000 permutations. From the changes in Figure 2B, one can observe the changes in the stability of data after *nperm* * 1000 disturbances.

## EXAMPLE APPLICATIONS

In this section, we provide two examples to demonstrate the effectiveness and applicability of the ccml method in multi-omics data integration.

### Example 1: the Karolinska COSMIC cohort of COPD

To demonstrate the utility of ccml, we applied it to multi-omics integrative clustering analysis of our Karolinska COSMIC cohort (www.clinicaltrials.gov/ct2/show/NCT02627872), designed to investigate sex differences in smoking-associated COPD [14–20]. We utilized nine omics data-blocks (mRNA, miRNA, proteomes, lipidomes and metabolomes) collected from several anatomical locations (see Figure 2) from 52 female subjects (20 healthy, 20 smokers, 12 COPD): mRNA from bronchoalveolar lavage (BAL) cells collected by microarray [19]; miRNA from BAL cells and from exosomes from BAL fluid (BALF) collected by microarray [19, 21]; difference gel electrophoresis proteomics from BAL cells [14]; shotgun proteomics data from BAL cells collected by isobaric tags for relative and absolute quantitation (iTRAQ) mass spectrometry (MS) [22, 23]; shotgun proteomics data from bronchial epithelial cells (BEC) collected by means of tandem mass tag–MS [24]; eicosanoid profiling data from serum and BALF [18]; and metabolomics data from serum [25]. There are 607 multi-omics combinations from single to 8-omics combinations with sample existing percentage (percent of samples tested in certain multi-omics combinations) ranging from 34.6 to 100%.

To identify molecular subgroups, we first applied SNF to all 607 multi-omics combinations and further conducted unsupervised clustering using the SC method. For each *n*-tuple omics combination, a fused subject-to-subject similarity matrix was calculated from the SNF analysis with different subject sizes as missing samples. The group membership was then predicted using SC for each *n*-tuple omics combination using the R-package SNF tool. The output of this step resulted in a 52-by-607 matrix of predicted labels, which was then inputted into the ccml framework. Within ccml, we evaluated two clustering methods (SC and hierarchical clustering, HC), two weights (NCW and CW) and two omics strategies (EQUAL for input of all *n*-omics clustering results and LARGER for all larger and equal to *n*-omics combinations). As the Karolinska COSMIC cohort consists of clinically well defined,relatively homogeneous groups , we evaluated accuracies of normalized mutual information (NMI) between true label with predictive clusters, as shown in Figure 2C. Our results showed that SC outperformed hierarchical clustering (*t*-test, *P* < 0.01).

To ensure adequate sample coverage, we selected a threshold of 40% for the existing sample rate, as depicted in Figure 2D. The clustering accuracy strongly correlated with the number of omics used (Pearson correlation coefficients *r* > 0.8, *P* < 0.01). Specifically, when integrating multi-omics combinations with ≥5 omics, the accuracy was twice that of single-omics prediction for 49 out of 52 subjects (Figure 2D). Figure 2E shows the accuracy changing with number of omics and the threshold of existing sample rates in SC of NCW with the LARGER strategy. Notably, the new NCW demonstrated robust performance.

### Example 2: the U-BIOPRED cohort of adult asthma

To further exemplify the utility of ccml in large-scale omics characterizations, we have extended our framework for multi-omics integrative subgrouping developed for the Karolinska COSMIC cohort to the U-BIOPRED cohort (https://europeanlung.org/en/projects-and-campaigns/past-projects/u-biopred/), designed to investigate molecular subgroups of severe asthma [26]. This framework integrates multi-level omics data from multiple anatomical locations using SNF, SC and ccml to identify distinct molecular subgroups of diseases [26–30]. The analysis, called the ‘molecular handprint’, utilizes 24 omics data-blocks collected from 498 asthma patients, including genotyping, mRNA, proteomes, metabolomes, breathomics, computed tomography from expiration and inspiration, metabolomics, microbiome and drugomics data.

As demonstrated by our analysis of the U-BIOPRED cohort, the proposed framework holds great potential in identifying robust molecular subgroups of asthma with clinical relevance. Our analysis of the 498 asthma samples utilizing 24 omics data-blocks resulted in the identification of three robust clusters, indicating that the accuracy of predictive clustering increases with the integration of more omics data (see Figure 2F). We measured accuracy by comparing predictive labels generated by ccml with the final predictive cluster labels. These findings not only contribute to a better understanding of the molecular mechanisms underlying asthma, but also underscore the potential of the molecular handprint framework in facilitating more personalized approaches to asthma treatment.

Overall, these two case studies provide compelling evidence of the effectiveness and versatility of the ccml approach for integrating multi-level omics data from different platforms and anatomical locations to identify molecular subgroups of diseases. This approach has the potential to significantly impact clinical research by enabling more personalized treatment approaches and improving patient outcomes. Future studies could expand upon this work by exploring the application of ccml to other disease contexts and evaluating its potential for translating molecular subtyping approaches into clinical practice.

## DISCUSSION

In this study, we introduced ccml, an innovative consensus clustering method designed to address the challenge of unequal sample coverage in the integrating of high-dimensional, heterogeneous datasets. As an open-source software for unsupervised class discovery, ccml enables multi-omics integrative prediction with unequal sample coverage, thus maximizing the information obtained from such integration and increasing sample coverage. The effectiveness of ccml in identifying robust molecular subgroups of diseases is demonstrated in case studies using the Karolinska COSMIC COPD cohort and the U-BIOPRED asthma cohort. These findings highlight the potential of ccml to uncover clinically meaningful and reproducible molecular subgroups.

Our study makes a valuable contribution to the field of multi-omics data integration by providing a novel approach to identify molecular subgroups in diseases. By integrating various omics data types and different levels of multi-omics combinations, ccml facilitates a more comprehensive understanding of the molecular mechanisms underlying complex diseases. Ultimately, this approach may lead to improved diagnosis, prognosis, and personalized treatment.

In addition, prediction accuracy may be influenced by cohort-specific characteristics, including the level of homogeneity of a clinical cohort, variations in the range of multi-omics combinations and the distribution of missing samples, which can vary significantly between different cohorts. This is demonstrated by the two example cohorts utilized in this paper: The Karolinska COSMIC cohort study design was aimed at selecting a specific subgroup of COPD patients and relevant controls to investigate molecular sex differences in early-stage COPD. As such, the inclusion and exclusion criteria were designed to create as homogeneous subgroups as possible, with no comorbidities or pharmaceutical treatments allowed, within a narrow age span to focus on post-menopausal women, carbon monoxide monitoring the day of sampling to control for the acute effects of smoking and all clinical samples collected at a single site by the same team to minimize technical variance. The COSMIC study was thus included to exemplify a focused study design, with homogeneous groups and known true labels. In contrast, the BIOPRED cohort was designed to investigate the full breadth of severe asthma, with broad inclusion criteria allowing for the full range of pharmaceutical treatments used for asthma, with a broad age span, sample collection at multiple sites spanning over multiple countries and cultures in Europe, and with some inconsistencies in the specific samples collected from each subject as well as the omics analyses performed from the collected samples. As such, the U-BIOPRED cohort was included to exemplify the use of the CCML tool in a population-based, complex study design without a priori defined subgroups.

Our findings also have potential implications for clinical research, particularly in the areas of precision medicine and biomarker discovery. The ability to identify robust molecular subgroups of diseases using ccml could enable the development of targeted therapies for specific patient subgroups, which can improve clinical outcomes and reduce healthcare costs [2].

In terms of future work, we plan to extend the ccml framework by including additional data types, such as imaging and clinical data, to further enhance the accuracy and clinical relevance of molecular subgroups. We also aim to apply ccml to other disease cohorts to assess its generalizability and reproducibility. Overall, ccml has the potential to make a substantial impact on the field of multi-omics data integration and contribute to the advancement of precision medicine.

**Key Points**

- The ccml workflow addresses limitations posed by missing data in multi-omics integration, which is inevitable in human cohorts consisting of multiple data modalities.
- The ccml package provides a novel means of adjustment to the consensus clustering algorithm based on permutation of the similarity matrix to establish the background significance level.
- Evaluation of the ccml package using the Karolinska COSMIC cohort demonstrates that the method can be used to facilitates multi-omics integration using data platforms with up to 60% missing data with equally robust—or improved—similarity in co-clustering of subjects compared to traditional methods.

## Author Biographies

**Chuan-Xing Li**, PhD, is an assistant professor at the Karolinska Institute (Sweden). Her research interest lies in computational precision medicine and multi-omics integration.

**Hongyan Chen** is a graduate student at the School of Biomedical Engineering, Wenzhou Medical University. Her research interests include deep learning and computational precision medicine.

**Nazanin Zounemat-Kermani**, PhD, is a postdoctoral researcher at the Data Science Institute and National Heart & Lung Institute, where she implements machine learning methods for the integration of multi-omics datasets for the endotyping of respiratory diseases.

**Ian M. Adcock**, PhD, is a professor of Respiratory Cell & Molecular Biology at Imperial College London. His research interests include regulation of airways inflammation by glucocorticoids and molecular sub-phenotyping of severe asthma and COPD using multi-omics integration.

**C. Magnus Sköld**, MD, PhD, is a professor of Respiratory Medicine at Karolinska Institutet and a senior consultant at Karolinska University Hospital, Stockholm, Sweden. Professor Sköld’s main research interests include clinical and translational studies on chronic obstructive pulmonary diseases and pulmonary fibrosis.

**Meng Zhou** is a professor at the School of Biomedical Engineering, Wenzhou Medical University. His research interests include bioinformatics, computational precision medicine and immuno-oncology.

**Åsa M. Wheelock**, PhD, is an associate professor and the head of the Respiratory Medicine Unit, Department of Medicine and Centre for Molecular Medicine at the Karolinska Institute, Stockholm, Sweden. Her research interests involve molecular sub-phenotyping of heterogeneous diagnoses of obstructive lung disease, such as COPD, asthma and post-acute sequelae of COVID-19 (PASC) using multi-omics integration and systems medicine approaches.
